# Evolution of Experience of Care of Patients with and without Chronic Diseases following a Québec Primary Healthcare Reform

**DOI:** 10.1155/2016/2497637

**Published:** 2016-04-07

**Authors:** Raynald Pineault, Roxane Borgès Da Silva, Sylvie Provost, Mylaine Breton, Pierre Tousignant, Michel Fournier, Alexandre Prud'homme, Jean-Frédéric Levesque

**Affiliations:** ^1^Direction de Santé Publique de l'Agence de la Santé et des Services Sociaux de Montréal, 1301 Rue Sherbrooke Est, Montréal, QC, Canada H2L 1M3; ^2^Institut National de Santé Publique du Québec, 190 Boulevard Crémazie Est, Montréal, QC, Canada H2P 1E2; ^3^Centre de Recherche du Centre Hospitalier de l'Université de Montréal, 900 Rue Saint-Denis, Montréal, QC, Canada H2X 0A9; ^4^Institut de Recherche en Santé Publique de l'Université de Montréal, 7101 Avenue du Parc, Montréal, QC, Canada H3N 1X9; ^5^Faculté des Sciences Infirmières de l'Université de Montréal, 2375 Chemin de la Côte-Sainte-Catherine, Montréal, QC, Canada H3T 1A8; ^6^Centre de Recherche de l'Hôpital Charles LeMoyne, 150 Place Charles-LeMoyne, Bureau 200, Longueuil, QC, Canada J5C 2B6; ^7^Département des Sciences de la Santé Communautaire, Université de Sherbrooke, 3001 12e Avenue Nord, Sherbrooke, QC, Canada J1H 5H3; ^8^Department of Epidemiology, Biostatistics and Occupational Health, McGill University, 1020 Avenue des Pins Ouest, Montréal, QC, Canada H3A 1A2; ^9^Centre for Primary Health Care and Equity, University of New South Wales, Level 3, AGSM Building, Sydney, NSW 2033, Australia; ^10^Bureau of Health Information, 67 Albert Avenue, Chatswood, NSW 2067, Australia

## Abstract

*Objectives*. To assess the extent to which new primary healthcare (PHC) models implemented in two regions of Quebec have improved patient experience of care, unmet needs, and use of services for individuals with and without chronic diseases, compared with other forms of PHC practices.* Methods*. In 2005 and 2010, we carried out population and organization surveys. We divided PHC organizations into new model practices and other practices and followed the evolution over time of patient experience of care.* Results*. Patients with chronic diseases had better accessibility but worse continuity of care in the new model practices than in the other practices at both time periods. Through the reform, accessibility decreased evenly in both groups, but continuity and perceived outcomes improved more in the other practices. Use of primary care services decreased more in the new model practices. Among patients without chronic disease, accessibility decreased much less in the new models and responsiveness increased more. There was no significant change in ER attendance and hospitalization.* Conclusion*. The evolution of patient experience of care has been more favorable for patients without chronic diseases. These findings raise concerns about equity since the aim of the PHC reform was targeting in priority individuals with the greatest needs.

## 1. Introduction

Increase in the number of the elderly and in the prevalence of chronic diseases presses healthcare systems to offer individuals with chronic diseases more comprehensive care through primary healthcare (PHC) [1–3]. To meet these needs, different models of chronic disease management have been proposed. The most widely known is the “Chronic Care Model” that advocates an integrated approach to care at all levels of the healthcare system for comprehensive and multidisciplinary management of chronic diseases [4]. It also advocates for the establishment of organizational mechanisms to support provision of services and interorganizational linkage to achieve better coordination and integration of services. Related to the “Chronic Care Model,” other models have been proposed for the delivery of primary healthcare, including the “Patient-Centered Care Medical Home” [5–9]. All proposed models focus on common elements, including sharing of responsibilities between healthcare professionals in a multidisciplinary teamwork perspective, active role of individuals in monitoring of their diseases, importance of communication facilitated by a clinical information system, and integration of services in an organizational framework that supports clinical practices and fosters linkages with other components of healthcare systems [3, 10].

Based on these models, two new forms of PHC practices were created in the early 2000s in Quebec: Family Medicine Groups (FMGs) aimed at improving continuity of care and Network Clinics (NCs) intended to provide greater accessibility of services. A FMG is composed of 6 to 10 physicians with no geographical catchment area for patients they can register (between 10,000 and 15,000 patients by FMG). The FMG can count on a grant from the Ministry of Health and Social Services to support its operations, in exchange for a contractual engagement to register a predetermined number of patients and to provide a minimum of specified services. It also provides greater accessibility through extended hours and participation in a regional on-call system. As of November 2010, there were 217 accredited FMGs, including 25 in Montréal and 35 in Montérégie, the two regions that participated in this study. The NC is a complementary PHC practice model implemented in both regions, but mainly in Montréal at the initiative of the Regional Health and Social Services Agency. It specifically aimed to improve accessibility, through providing walk-in services and ensuring access to radiology and laboratory tests as well as medical specialist services. It receives financial support from the Regional Agency. In August 2010, there were 33 NCs in Montréal and 4 in Montérégie. The distinction between FMG and NC is often blurred, especially as some PHC practices have a dual FMG-NC status, thus benefiting from two sources of funding. The complementarity between FMGs and NCs was to allow the provision of more complete and better integrated care, particularly for individuals with chronic diseases [11–13]. In November 2010, 19 PHC practices had a FMG-NC status in Montréal and none in Montérégie.

Several studies have explored the association between structural features of PHC practices and experience of care [14–18]. Studies have also assessed the impact of implementing specific components of the Chronic Care Model on various care processes and outcomes [19–21]. Few studies have looked at PHC practice organizational attributes to assess their potential for managing chronic diseases [3, 4]. An Ontario study found that chronic disease management was superior in Community Health Centers compared to other types of practices mainly due to the presence of nurse practitioners and interdisciplinary teams [22]. In the context of a large study conducted in 2005 in two Quebec regions, we examined the overall patient experience of care in PHC practices and found a better continuity of care among practices that were more integrated and coordinated, but the model that focused on walk-in visits provided more accessible care [13, 23]. This study reported on the situation prevailing in 2005 and did not address the impact of the new PHC practices as it was too early to assess the change. A related cohort study found that FMGs represented an integrated model of PHC delivery associated with higher levels of achievement in chronic care [24].

In close collaboration with the Regional Health and Social Services Agencies of the two most heavily populated Quebec regions, Montréal and Montérégie, we conducted a second study in 2010 to assess the evolution of the PHC reform and its impacts on patients' experience of care, unmet needs, and use of services [25].

The main objective of this paper is to assess the extent to which new PHC practice models implemented in the two regions (FMG-NC, FMG, and NC) have improved patient experience of care, unmet needs, and use of services for individuals with and without chronic diseases, compared with other forms of PHC practices that did not undergo such a change.

## 2. Methods

### 2.1. Design

**Figure 1:**
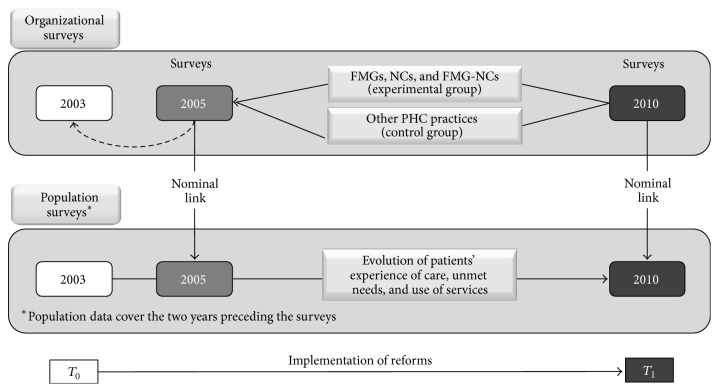
Study design.

The study design corresponds to a before-after natural experiment in which FMGs, NCs, and FMG-NCs constituted the experimental group and the other practices formed the control group (Figure 1). The study consisted of population and organization surveys conducted in 2005 and 2010 in Montréal and Montérégie [25]. At that time, the two regions were divided into 23 local territories (12 in Montréal and 11 in Montérégie) under the governance of Health and Social Services Centres. The population surveys were carried out on independent samples of the 2005 and 2010 populations. They included 9,206 adults aged 18 or more in 2005 with a response rate of 64% and 9,180 in 2010 with a response rate of 56%. For the purpose of this study, we excluded respondents who did not have a regular source of care since only those who had a regular source of care reported on their experience of care. We also excluded those whose usual source of care was not identified at both time periods. These exclusions left 6,198 respondents in 2005 and 6,753 in 2010. Short telephone surveys of all PHC organizations were also carried out: 659 organizations in 2005 and 606 organizations in 2010. Basic information such as type of practice (solo, group, FMG, and NC), number of practicing family physicians, having a nurse in the clinic, and offering walk-in services was gathered through telephone calls to the receptionists of all practices.

Population survey samples were stratified with approximatively 400 respondents in each of the 23 local territories regardless of their total population. The questionnaire was constructed drawing mainly on two validated instruments: the Primary Care Assessment Survey and the Primary Care Assessment Tool, to which we added questions when the topic had not been addressed [26–28]. It focused on respondents' attachment to a PHC practice, experience of care, and use of services in the two years preceding the surveys as well as unmet needs in the six months preceding the survey. The population questionnaire documented also individual characteristics such as gender, age, level of education, economic status, perceived health, and presence of morbidities [25].

Population surveys data were linked with information gathered on PHC organization through respondents' identification of their regular source of primary care in the two years preceding the survey (2003–2005 and 2008–2010). Failing to precisely identify a usual source of primary care, respondents identified the PHC practice most frequently attended in the two years preceding the survey. This practice was considered the respondent's usual source of care.

### 2.2. Organizational Variables

PHC organizations were classified into two groups: an experimental group that included new model practices (FMGs, NCs, and FMG-NCs) and a control group that included all other practices (Local Community Services Centres (LCSCs), Family Medicine Units (FMUs), and solo and group practices that were not FMG or NC). FMUs are academic training units that are likely to reflect current practice of family medicine and thus espouse hosting practices' dominant philosophy of care. For the purposes of this study, FMGs implemented in LCSC and FMU were included in the experimental group. These represented 11 LCSCs out of 49 and 9 FMUs out of 12.

In 2010, among the different PHC organization models in Montréal and Montérégie, group and solo practices that were not FMG or NC were dominant, as they represented 73% of all organizations and were the usual source of care for 50% of all service users. Regardless of their status (FMG, NC, or none), they are privately run by physicians, who are paid on a fee-for-service basis. Costs are shared, but not income.

### 2.3. Patients' Experience of Care, Use of Services, and Unmet Needs

Information concerning patients' experience of care, use of services, and unmet needs was gathered from population surveys. Experience of care and utilization referred to the two years preceding the survey and unmet needs to the six months preceding the survey. Variables of experience of care were accessibility, continuity, comprehensiveness, responsiveness, and perceived care outcomes [29]. Operationalization details for these variables are presented in Table 8.

The 28 selected indicators of experience of care were grouped under five dimensions: accessibility (6 items), continuity (5 items), comprehensiveness (5 items), responsiveness (7 items), and perceived care outcomes (5 items). We carried out factor analysis within each of the five dimensions and calculated Cronbach's alpha which reached values of 0.60 or more for continuity (0.61), comprehensiveness (0.79), responsiveness (0.63), and care outcomes (0.82) but was low for accessibility (0.30), presumably reflecting the formative nature of this index [30]. Accessibility items are not highly correlated. This characterizes a composite index rather than a reflective scale [31]. Variables of experience of care were expressed as scores on a 10-point scale. Utilization of services and unmet needs were dichotomous variables.

### 2.4. Selection of Chronic Diseases

Data on chronic diseases come from population surveys. Respondents were asked if a doctor had told them they were suffering from specific chronic diseases. We then classified chronic diseases as follows: heart disease (coronary artery disease, heart failure), respiratory disease (chronic obstructive pulmonary disease, asthma), musculoskeletal disease (arthritis, osteoarthritis, and rheumatism), cardiometabolic diseases (hypertension, diabetes, and hypercholesterolemia), and other chronic diseases which was a residual and more heterogeneous category that included all remaining chronic diseases such as cancer, anemia, and gastrointestinal disorders. Mental illnesses were not excluded; they were considered as comorbidities associated with all the above categories, including the no chronic disease one; to be included in the broad category “with chronic disease,” respondents had to have at least one chronic disease listed above. All others were included in the no chronic disease category.

### 2.5. Data Analysis

To test for differences in the evolution of experimental and control groups between 2003 and 2010, we applied the difference-in-differences (DD) technique and matched individuals with the propensity score method to ensure better comparability of the two groups [32–36]. This method is particularly well suited to compare change in outcomes over time between individuals exposed to an intervention (the experimental group) and individuals that were not (control group) [34]. Individuals were matched on the basis of propensity scores that estimate the probability for an individual with given characteristics to be attached to a PHC organization of the experimental group [37]. The propensity score acts as a balancing score that renders the distribution of observed baseline covariates similar between the experimental group and the control group [38]. The propensity score was calculated with logistic regression using sex, age, level of education, economic situation, immigration status, perception of health status, and morbidities as predictors.

The subjects were then matched applying the Kernel method, in which treated subjects are matched with a weighted average of all controls with weights that are inversely proportional to the distance between the propensity scores of treated individuals and controls. As the groups were very similar at the onset, nearly all subjects could be correctly matched. We carried out analyses using STATA (version 13). Difference-in-differences analyses were conducted separately on respondents with and without chronic diseases. Effect size, which expresses the magnitude of change, was measured in percentage and calculated by dividing DD scores by the average value of the indicators in the experimental and control groups at the baseline time period.

## 3. Results

### 3.1. Descriptive Results

Table 1 presents the percentage of respondents with a regular source of primary care. As expected, the percentage is higher for those with than for those without chronic diseases at both time periods. For both groups, the percentages significantly increased between 2003–2005 and 2008–2010.

Table 2 shows, among those who had a regular source of primary care, percentages of users with different chronic diseases in the 2005 and 2010 surveys.

Table 3 presents for each category of chronic diseases in the two time periods the percentage of those who, aside from that disease, also present other chronic morbidities. Cardiac and musculoskeletal diseases have a higher percentage of associated multiple chronic morbidities. Data for the two time periods are generally comparable except for “cardiometabolic” and “other chronic diseases.”

Tables 4 and 5 present data on other users' characteristics for differences between control and experimental groups of the two samples. There are very few differences that are statistically significant.

### 3.2. Evolution of Experience of Care, Use of Services, and Unmet Needs

The impact of the introduction of new PHC models for individuals with chronic diseases was tested by comparing, in 2003–2005 and 2008–2010, individuals attached to FMG-NC, FMG, and NC (experimental group) to those attached to other practices that had not changed (control group) with regard to experience of care, unmet needs, and use of services. As shown in Table 6, accessibility presents the lowest score among indices of experience of care. It decreased in both control and experimental groups over the years, but the experimental group remained with higher score and statistically significant differences at both times (*p* = 0.048; *p* = 0.004). However, the change was not sufficient to yield a significant DD value (*p* = 0.640). Continuity was high in both groups. At both times, scores were higher for the control group, and the difference even increased in 2008–2010 (*p* = 0.001; *p* < 0.001). The DD value approached the 0.05 level of significance (*p* = 0.08) with a small negative effect size of −1.5%. The pattern for comprehensiveness and responsiveness is very similar to that of continuity with nonsignificant DD values. Care outcomes show higher scores for the control group with increased differences in 2008–2010. This resulted in a significant DD value of −1.6 in favor of the control group (*p* = 0.044) and an effect size of −1.8%. For unmet needs and utilization indicators, the results show no significant results except for high utilization of the usual source of primary care that decreased in both groups, but more so in the experimental group (−7.9) than in the control group (−4.0), resulting in a significant and negative DD value (*p* = 0.049), corresponding to a 12.7% decrease.

We carried out similar analyses for respondents with no chronic disease. Data presented in Table 7 show overall decline in accessibility over time in both experimental and control groups. However, the decline was larger in the control than in the experimental group while it was very small in the experimental group, yielding a positive and significant DD value (*p* < 0.001) with a 5.5% increase. As regards continuity, the differences between groups remain constant over time in favor of the control group and this lack of change resulted in a nonsignificant DD value (*p* = 0.563). The difference between groups for responsiveness turned in favor of the experimental group in 2008–2010, resulting in a positive and significant DD value (*p* = 0.007) with a positive 2.3% change. Aside from these results, the evolution in both groups for all other indicators has been comparable, with no significant DD values.

## 4. Discussion

Patients with chronic diseases in the experimental group had a worse experience of care than those in the control group in 2008–2010 on all indicators, except accessibility that was better and responsiveness that showed no difference. The percentage of users who reported unmet needs was lower in the control group and hospitalization was slightly higher in the experimental group, but these differences were not significant.

Looking at the evolution of experience of care for patients with chronic diseases in the two groups, measured by DD values, we observed that continuity increased more in the experimental than in the control group although the DD value failed to reach the 0.05 level of significance (*p* = 0.081). Perceived care outcomes increased more in the control than in the experimental group resulting in a negative and significant DD value. The percentage of users attending more often their usual source of primary care also declined more in the experimental than in the control group and these results are statistically significant (*p* = 0.049) with a percentage of change close to 13%.

The findings for patients with no chronic disease contrast with those of patients with chronic diseases. In 2008–2010, accessibility was better in the experimental group than in control group (*p* < 0.001), but continuity was worse (*p* < 0.001). There was no other significant result. DD values are positive and statistically significant for accessibility with a 5% effect size, indicating less unfavorable evolution for the experimental group (*p* < 0.001). Another significant and positive DD value is for responsiveness (*p* = 0.007). Continuity shows no difference between groups in its evolution, nor with all the other indicators of experience of care, unmet needs, and utilization.

These findings raise concern about expected benefits resulting from Quebec healthcare reforms. First, it is disappointing to see that while continuity improved in the population it did so more in the control than in the experimental groups. The main objective pursued by the reform in providing additional resources to new model practices was precisely to improve continuity of care, especially among individuals with chronic diseases [11, 12]. A possible explanation for this result could be that financial incentives to enrollment of patients with chronic diseases were extended to all PHC organizations, thus offsetting the advantage enjoyed up to that point by FMGs. Another explanation may be increased provision of services by nurses in FMGs and NCs that are not included in our measures of continuity. Our measures of experience of care targeted mainly services rendered by physicians. Consequently, they may underestimate services rendered by professionals other than doctors.

These findings regarding continuity do not support those of an earlier study conducted by Tourigny et al. among 1,275 patients followed up in five FMGs [39]. That study found an increase in continuity among patients 18 months after their enrollment in FMGs. However, Tourigny et al.'s study did not have a control group and was limited to only five FMGs.

A second unanticipated result concerns accessibility which showed less unfavorable evolution for patients without chronic diseases in the experimental group than for patients with chronic diseases. Again, the establishment of new models of PHC organizations, and particularly of NCs, was aimed at improving accessibility to care for the general population, but in priority to the neediest individuals. These included patients with chronic diseases. It is rather ironic that the ones more in need were more affected by the unfavorable evolution of accessibility.

Another finding that raises concerns is the lack of impact of the reform on unmet needs and use of services. Conversely, the larger decrease in the percentage of the high users of services at the usual source of primary care in the experimental group can be considered as a positive outcome, possibly associated with more comprehensive and preventive care. It could also result from substitution between services provided by physicians and those provided by nurses or other health professionals. We had no data to support these plausible explanations. However, the impact of this outcome remains marginal if not coupled to decrease in ER attendance and hospitalization that represent the most costly services.

Our findings regarding accessibility and utilization corroborate studies reporting on FMGs. In an ecological study of the entire Quebec population, Dunkley-Hickin found that a higher degree of physicians' participation in FGM in a geographical area was not associated with improved accessibility among users of services living in that area [40].

In a large scale cohort study involving 79 FMGs, Strumpf observed no improvement in accessibility but a slight reduction in use of primary care and specialist services [41]. Based on the same administrative data banks, Héroux et al. followed up a cohort of 122,722 patients enrolled in FMGs compared to 675,102 who were not [42]. They found a small reduction in number of emergency visits for those attached to FMGs, but, as in our study, no change in hospitalization.

None of those studies has reported specifically on patients with chronic diseases compared to those without chronic diseases. In an earlier study conducted on the 2005 sample, we explored the relationship between models of PHC organizations and experience of care. We concluded that the “coordination-integrated model,” which matched the attributes of the ideal type of FMGs that were to be implemented at that time, emerged as the model with the greatest potential to concomitantly achieve high levels of accessibility and continuity for patients with chronic diseases [13]. Obviously, this prediction has not yet come true. It could be that the reform had an effect on both PHC organizations that have become FMGs or NCs and those that have not changed their status. It was not possible with our data to test that spillover effect hypothesis.

According to Rothman and Wagner, the future of primary healthcare largely depends on ability to improve healthcare delivery to meet the needs of chronically ill patients [3]. The Chronic Care Model provides six interrelated components for achieving such change among which is delivery system redesign [4]. This last condition does not seem to have been met in the implementation of new PHC models in Quebec.

A recent report of the Auditor General of Quebec to the National Assembly casts a critical look at the implantation of FMGs and NCs [43]. It explains the relative failure of these PHC organizations to fully attain the objectives established by the Ministry of Health and Social Services by many factors, notably the absence of clear rules, guidelines, and incentives and, above all, the lack of control of the Ministry and the Regional Agencies on the process of implementation. This means that several new PHC practices received complementary funding without respecting all the components of the contractual engagement.

Along the same lines, a recent report of the C.D. Howe Institute underlines that important factors associated with the slow implementation of FMGs are the low registration rate of patients and the deficient development of teamwork [44]. The findings of our study concur with those of other studies and support concerns expressed by analysts.

## 5. Limitations

Our study has limitations. The use of difference-in-differences (DD) method for isolating the specific effect of the reform is fully justified because it provides good internal validity. However, when carried out in a complex system such as the healthcare system, DD analyses cannot completely rule out the possibility of spillover effects. For example, it is possible that some aspects of the healthcare policies spilled over and influenced PHC practices of the unexposed control group or that new PHC model practices exerted a mimetic influence on the other practices [45].

Surveys lend themselves to possible recall bias by respondents. Concerning experience of care, unmet needs, and use of services, if present, biases should be equally distributed among respondents. Recall biases are less likely to occur when reporting events extending over a certain period of time rather than single events taking place at a given point in time.

Another limitation is self-reporting of chronic morbidities. Responses are always limited by respondents' subjective understanding and their capacity to report accurately medical information. The wording of the questions attenuated this possible effect by referring to validation of the diagnosis by a doctor (i.e., “has a doctor ever told you that you have diabetes?”). Categories of chronic diseases were defined and operationalized in the same way in both 2005 and 2010 surveys. However, for the category “other chronic diseases,” it is possible that our redefinition and classification were more inclusive in 2005, which may explain the higher percentage of respondents reporting this type of morbidity in 2005.

As pointed out in Methods, mental illnesses were not excluded but left in all categories of physical chronic conditions, including the no disease category. Many reasons justified our decision. The questions concerning mental health problems were slightly different in 2005 and 2010 as well as the time period reference: “have you ever had…” in 2005 and “in the last 2 years, did you see your doctor…” in 2010. This may explain the higher percentage of individuals reporting mental health problems in 2005 than in 2010. Aside from this difference between the two surveys, mental health problems happened to be evenly distributed among our categories of chronic physical diseases, including the no disease category. Consequently, we thought that their inclusion would minimize potential biases while increasing statistical power.

Measures of experience of care reflect patients' point of view and perceptions. They are subjective but still most appropriate to use in a patient-centered perspective. Our questionnaire largely draws from two validated instruments (PCAS and PCAT) [26–28]. We constructed new scales of experience of care, more adapted to our context. Following the classical theory of measurement, we carried out factor analysis and calculated Cronbach's alpha coefficients which have values close to the commonly accepted level (0.70) except for accessibility (0.30) [46]. We still considered accessibility a valid measure based on the formative approach to measurement. In contrast to the classical reflective approach, in the formative approach items composing an index are not necessarily correlated or substitutable with each other, as is the case in reflective scales [30, 31].

As mentioned earlier, our scales of experience of care may fail to include processes of care rendered by health professionals other than doctors. In that sense, they may underestimate experience of care in contexts of multidisciplinary work and thus fail to fully account for the effect of introducing new types of professionals, as is the case in FMGs and NCs. The possibility of such a bias has been suggested, particularly by researchers using large administrative data banks based on physicians' reimbursement claims [47, 48].

The 2003–2005 baseline time period was very close to the onset of the reform. The first FMGs were accredited in March 2003. Since questions related to experience of care and use of services referred to the two years preceding the population surveys, we are confident that the 2003–2005 time period was a valid baseline in the context of a natural experiment. Furthermore, as expected, changes took some time before being fully implemented in PHC practices after their accreditation.

## 6. Conclusion

Our study's findings raise questions concerning benefits resulting from the introduction of new PHC practice models in Québec. The aim of the PHC reform was mainly to improve accessibility and continuity of care for the entire population, but especially for individuals in greater needs such as those with chronic diseases. Our findings show that, for patients with chronic diseases attached to new PHC model practices, continuity increased less than for those attached to other practices. Accessibility decreased for all users of services but much less for those with no chronic disease in the new model PHC practices but did not change for those with chronic diseases. These results raise equity concerns since the ones with more needs seem to have benefitted less from the reform than those less in needs. Furthermore, the reform did not reduce the use of the most costly services, ER attendance, and hospitalization, nor did it reduce the reporting of unmet needs. Based on those findings, it is difficult to conclude that the Québec healthcare reform has been so far successful.

## Figures and Tables

**Table 1:** Percentage of respondents having a regular source of PHC according to the presence of chronic disease, 2003–2005 and 2008–2010.

	2003–2005	2008–2010	*p* ^*∗*^
At least one chronic disease	(*n* = 4870)	(*n* = 5424)	
	79.1	86.5	<0.001

No chronic disease	(*n* = 4336)	(*n* = 3756)	
	63.7	71.4	<0.001

^*∗*^Difference of proportions test.

**Table 2:** Percentage of users with various chronic diseases, 2003–2005 and 2008–2010.

	2003–2005	2008–2010	*p* ^*∗*^
	(*n* = 6198)	(*n* = 6753)	
At least one chronic disease	58.9	64.4	<0.001
Cardiac	*7.4*	*9.4*	<0.001
Respiratory	*14.6*	*15.3*	0.287
Musculoskeletal	*23.4*	*26.0*	<0.001
Cardiometabolic	*24.0*	*33.4*	<0.001
Other chronic diseases	*31.2*	*25.9*	<0.001

^*∗*^Difference of proportions test.

**Table 3:** Percentage of users having a chronic disease associated with multiple morbidities, 2003–2005 and 2008–2010.

	Cardiac			Respiratory			Musculoskeletal			Cardiometabolic			Other chronic diseases		
	2003–2005	2008–2010	*p* ^*∗*^	2003–2005	2008–2010	*p* ^*∗*^	2003–2005	2008–2010	*p* ^*∗*^	2003–2005	2008–2010	*p* ^*∗*^	2003–2005	2008–2010	*p* ^*∗*^
	(*n* = 459)	(*n* = 634)		(*n* = 904)	(*n* = 1030)		(*n* = 1452)	(*n* = 1755)		(*n* = 1486)	(*n* = 2253)		(*n* = 1934)	(*n* = 1750)	
	%	%		%	%		%	%		%	%		%	%	
Cardiac	—	—	—	13.3	14.2	0.566	13.9	15.8	0.139	19.7	20.3	0.672	15.7	13.5	0.061
Respiratory	26.1	23.0	0.237	—	—	—	20.9	20.6	0.867	15.3	16.3	0.407	20.9	18.2	0.038
Musculoskeletal	44.0	43.7	0.917	33.5	35.1	0.452	—	—	—	41.9	39.9	0.208	37.1	34.6	0.122
Cardiometabolic	63.5	72.1	0.004	25.1	35.6	<0.001	42.9	51.2	<0.001	—	—	—	34.7	38.8	0.011
Other chronic diseases	66.0	37.2	<0.001	44.7	30.9	<0.001	49.4	34.5	<0.001	45.2	30.1	<0.001	—	—	—

^*∗*^Difference of proportions test.

**Table 4:** Percentage (%) of users having at least one chronic disease according to selected characteristics, 2003–2005 and 2008–2010.

		2003–2005			2008–2010		
		Control	Experimental	*p* ^*∗*^	Control	Experimental	*p* ^*∗*^
		(*n* = 1831)	(*n* = 1770)		(*n* = 2198)	(*n* = 2151)	
Sex	Male	33.8	34.3	0.732	39.1	39.2	0.916
	Female	66.2	65.7		60.9	60.8	

Age	18–29	11.3	9.3	0.050	4.7	5.0	0.709
	30–44	21.0	23.2	0.113	13.6	16.5	0.007
	45–64	43.5	42.8	0.645	47.5	46.4	0.505
	65 or more	24.2	24.8	0.700	34.3	32.1	0.135

Level of education	No diploma	19.5	23.3	0.005	18.5	21.4	0.018
	High school diploma	32.7	34.9	0.174	31.3	32.1	0.538
	Collegial diploma	24.2	22.0	0.115	19.6	19.7	0.932
	University diploma	23.5	19.8	0.006	30.7	26.8	0.005

Economic status	Lowest	13.4	12.7	0.518	12.2	12.0	0.841
	Medium-low	32.1	34.7	0.101	31.8	31.8	0.975
	Medium-high	29.8	30.0	0.878	33.7	35.7	0.158
	Highest	24.7	22.7	0.141	22.3	20.5	0.130

Immigration status	Born in Canada	84.2	91.2	<0.001	84.1	88.8	<0.001
	Imm. <10 years	2.8	1.5	0.018	1.7	2.3	0.131
	Imm. ≥10 years	13.1	7.3	<0.001	14.2	8.9	<0.001

Perceived health	Bad/average	25.2	26.6	0.346	23.7	23.2	0.695
	Good	34.2	33.9	0.854	33.7	34.4	0.632
	Very good	28.9	28.3	0.671	31.2	30.1	0.457
	Excellent	11.7	11.2	0.676	11.5	12.3	0.384

Chronic disease	Cardiac	12.8	12.7	0.951	13.0	16.2	0.002
	Respiratory	24.0	26.2	0.131	24.4	22.9	0.241
	Musculoskeletal	39.3	41.4	0.214	39.6	41.1	0.323
	Cardiometabolic	41.9	40.6	0.401	52.7	50.9	0.241
	Other chronic diseases	53.3	54.1	0.622	39.8	40.7	0.518

^*∗*^Difference of proportions test.

**Table 5:** Percentage (%) of users without a chronic disease according to selected characteristics, 2003–2005 and 2008–2010.

		2003–2005			2008–2010		
		Control	Experimental	*p* ^*∗*^	Control	Experimental	*p* ^*∗*^
		(*n* = 1304)	(*n* = 1293)		(*n* = 1158)	(*n* = 1246)	
Sex	Male	41.6	43.3	0.368	43.2	42.6	0.781
	Female	58.4	56.7		56.8	57.4	

Age	18–29	26.5	25.5	0.587	15.1	12.4	0.050
	30–44	44.0	42.3	0.378	36.0	43.1	<0.001
	45–64	27.2	28.7	0.404	42.1	38.0	0.041
	65 or more	2.3	3.5	0.073	6.8	6.6	0.813

Level of education	No diploma	6.9	9.4	0.022	7.7	8.0	0.757
	High school diploma	31.4	35.0	0.047	28.5	27.2	0.481
	Collegial diploma	27.1	28.0	0.628	22.8	23.7	0.611
	University diploma	34.6	27.6	<0.001	41.0	41.1	0.971

Economic status	Lowest	9.4	8.2	0.297	6.9	7.4	0.652
	Medium-low	31.0	30.2	0.682	27.9	27.6	0.876
	Medium-high	33.4	34.0	0.718	35.2	34.8	0.805
	Highest	26.3	27.5	0.480	30.0	30.3	0.876

Immigration status	Born in Canada	77.2	84.2	<0.001	78.6	82.5	0.015
	Imm. <10 years	9.1	6.8	0.047	7.3	6.1	0.257
	Imm. ≥10 years	13.7	9.0	<0.001	14.2	11.4	0.042

Perceived health	Bad/average	7.2	6.5	0.473	5.2	6.7	0.107
	Good	22.8	22.7	0.944	23.5	24.2	0.701
	Very good	39.1	39.9	0.678	39.4	39.1	0.883
	Excellent	30.9	30.9	0.986	32.0	30.0	0.305

^*∗*^Difference of proportions test.

**Table 6:** Difference in differences (DD) of experience of care, unmet needs, and use of services (%) between users with at least one chronic disease who have FMG-NC, FMG, or NC as usual source of care and those who have another type of medical clinic, 2003–2005 and 2008–2010.

		2003–2005				2008–2010					
	Control group	Experimental group	Diff.	*p*	Control group	Experimental group	Diff.	*p*	DD	*p*	% of change
	(*n* = 1831)	(*n* = 1770)			(*n* = 2198)	(*n* = 2151)					
Accessibility of services	7.16	7.27	0.11	0.048	6.73	6.88	0.15	0.004	0.04	0.640	0.6
Continuity	8.58	8.39	−0.19	0.001	8.98	8.66	−0.32	<0.001	−0.13	0.081	−1.5
Comprehensiveness	8.62	8.52	−0.10	0.110	8.56	8.34	−0.22	<0.001	−0.12	0.184	−1.4
Responsiveness	8.82	8.71	−0.11	0.007	8.90	8.86	−0.04	0.277	0.07	0.202	0.8
Perceived care outcomes	8.75	8.65	−0.10	0.119	8.93	8.67	−0.26	<0.001	−0.16	0.044	−1.8
% of users reporting unmet needs for care	17.7	18.5	0.80	0.497	14.3	16.2	1.90	0.080	1.10	0.499	6.1
% of users who attended usual source of primary care six times or more	29.5	32.3	2.80	0.060	25.5	24.4	−1.10	0.394	−3.90	0.049	−12.7
% of users who attended emergency room at least once	38.4	41.2	2.80	0.088	37.7	39.1	1.40	0.321	−1.40	0.550	−3.5
% of users hospitalized at least once	20.2	21.2	1.00	0.502	21.2	23.3	2.10	0.085	1.10	0.510	5.3

**Table 7:** Difference in differences (DD) of experience of care, unmet needs, and use of services (%) between users without a chronic disease who have FMG-NC, FMG, or NC as usual source of care and those who have another type of medical clinic, 2003–2005 and 2008–2010.

		2003–2005				2008–2010					
	Control group	Experimental group	Diff.	*p*	Control group	Experimental group	Diff.	*p*	DD	*p*	% of change
	(*n* = 1304)	(*n* = 1293)			(*n* = 1158)	(*n* = 1246)					
Accessibility of services	7.32	7.25	−0.07	0.301	6.81	7.14	0.33	<0.001	0.40	<0.001	5.5
Continuity	7.73	7.48	−0.25	0.003	8.09	7.77	−0.32	<0.001	−0.07	0.563	−0.9
Comprehensiveness	8.40	8.31	−0.09	0.265	8.18	8.14	−0.04	0.628	0.05	0.663	0.6
Responsiveness	8.61	8.49	−0.12	0.022	8.78	8.86	0.08	0.127	0.20	0.007	2.3
Perceived care outcomes	8.39	8.29	−0.10	0.208	8.46	8.42	−0.04	0.575	0.06	0.630	0.7
% of users reporting unmet needs for care	19.3	18.8	−0.50	0.763	18.8	16.8	−2.00	0.194	−1.50	0.474	−7.9
% of users who attended usual source of primary care six times or more	12.9	12.8	−0.10	0.962	11.1	10.7	−0.40	0.717	−0.30	0.822	−2.3
% of users who attended emergency room at least once	30.1	30.4	0.30	0.841	31.4	31.1	−0.30	0.840	−0.60	0.776	−2.0
% of users hospitalized at least once	9.9	9.8	−0.10	0.909	12.7	12.4	−0.30	0.848	−0.20	0.955	−2.0

**Table 8:** Construction of experience of care indices.

The following questions refer to the usual PHC source identified by the respondents		
Accessibility of services		
If the doctor who is responsible for your care is not available, you can see another doctor?	0. Never/sometimes/often	1. Always
How long does it take to see the doctor by appointment?	0. Two weeks or more	1. Less than two weeks
How long does it usually take to get there?	0. 15 minutes or more	1. Less than 15 minutes
The office hours are convenient?	0. Not at all/a little/somewhat agree	1. Strongly agree
It is easy to reach someone by telephone to make an appointment?	0. Not at all/a little/somewhat agree	1. Strongly agree
It is easy to talk to a doctor or nurse by telephone?	0. Not at all/a little/somewhat agree	1. Strongly agree
Continuity		
You see the same doctor?	0. Never/sometimes/often	1. Always
How long have you been going there?	0. Five years or less	1. More than 5 years
Your medical history is known?	0. Not at all/a little/somewhat agree	1. Strongly agree
They are aware of all the prescribed drugs you take?	0. Not at all/a little/somewhat agree	1. Strongly agree
You can receive routine ongoing care for a chronic problem?	0. Not at all/a little/somewhat agree	1. Strongly agree
Comprehensiveness		
All your health problems are taken care of whether they are physical or psychological?	0. Not at all/a little/somewhat agree	1. Strongly agree
	
The doctor takes the time to talk to you about prevention and asks you about your lifestyle habits?	0. Not at all/a little/somewhat agree	1. Strongly agree
They help you get all the health care services you need?	0. Not at all/a little/somewhat agree	1. Strongly agree
Your opinion and what you want are taken into account in the care that you receive?	0. Not at all/a little/somewhat agree	1. Strongly agree
You are given help to weigh the pros and cons when you have to make decisions about your health?	0. Not at all/a little/somewhat agree	1. Strongly agree
Responsiveness		
How long do you have to wait between the scheduled time of appointment and the time you actually see the doctor?	0. Less than 60 minutes	1. 60 minutes or more
The staff answer your questions clearly ?	0. Not at all/a little/somewhat agree	1. Strongly agree
You feel respected?	0. Not at all/a little/somewhat agree	1. Strongly agree
You are greeted courteously at the reception?	0. Not at all/a little/somewhat agree	1. Strongly agree
Your physical privacy is respected?	0. Not at all/a little/somewhat agree	1. Strongly agree
The doctors spend enough time with you?	0. Not at all/a little/somewhat agree	1. Strongly agree
The local of the clinic are pleasant?	0. Not at all/a little/somewhat agree	1. Strongly agree
Perceived care outcomes		
The services you get help you to better understand your health problems?	0. Not at all/a little/somewhat agree	1. Strongly agree
The services you get help you to prevent certain health problems before they appear?	0. Not at all/a little/somewhat agree	1. Strongly agree
The services you get help you to control your health problems?	0. Not at all/a little/somewhat agree	1. Strongly agree
The professionals you see encourage you to follow the treatments prescribed?	0. Not at all/a little/somewhat agree	1. Strongly agree
The professionals you see help motivate you to adopt good lifestyle habits?	0. Not at all/a little/somewhat agree	1. Strongly agree
Unmet needs		
During the last 6 months, did you feel you needed to see a doctor for a health problem but didn't see one?	0. No	1. Yes

## Additional Points

Experience of care was not better in 2010 among patients with chronic diseases in new PHC practices models. Evolution of experience of care was not more favorable among these patients than those in the other practices. For patients with no chronic disease and those attached to the new PHC practice models, accessibility increased more than for patients with chronic diseases. So far, the benefits resulting from the reform have been less than expected.
